# Prognostic Impact of Memory CD8(+) T Cells on Immunotherapy in Human Cancers: A Systematic Review and Meta-Analysis

**DOI:** 10.3389/fonc.2021.698076

**Published:** 2021-06-25

**Authors:** Yao Jin, Aili Tan, Jia Feng, Zexi Xu, Peiwei Wang, Peng Ruan, Ruijun Luo, Yiming Weng, Min Peng

**Affiliations:** ^1^ Department of Oncology, Renmin Hospital of Wuhan University, Wuhan, China; ^2^ Department of Obstetrics & Gynecology, Renmin Hospital of Wuhan University, Wuhan, China

**Keywords:** human cancers, memory CD8(+) T cell, immunotherapy, immune checkpoint, prognosis, meta-analysis

## Abstract

**Objective:**

The objective of this systematic review and meta-analysis was to determine the prognostic value of memory CD8(+) T cells in cancer patients with immunotherapy.

**Methods:**

EMBASE, MEDLINE (PubMed), and Web of Science databases were searched to identify suitabile articles published before March 2021. Risk of bias on the study level was assessed using the Cochrane Bias Risk Assessment Tool. The hazard ratios (HRs) and 95% confidence intervals (CIs) of pooled progression-free survival (PFS) and overall survival (OS) were calculated using RevMan 5.4 to evaluate the prognostic impact of memory CD8(+) T cells.

**Results:**

In total, nine studies were included in the final analysis. High levels of memory CD8(+) T cells were significantly closely correlated with better progression-free survival (PFS) and overall survival (OS) of cancer patients with immunotherapy (PFS, HR 0.64, 95% CI 0.53–0.78; OS, HR 0.37, 95% CI 0.21–0.65). Memory CD8(+) T cells still have significant prognostic value in cancer patients given immunotherapy alone after excluding of other interfering factors such as chemotherapy, radiotherapy, and targeted therapy (PFS, HR 0.65, 95% CI 0.48–0.89; OS, HR 0.23, 95% CI 0.13–0.42). However, high memory CD8(+) T cells levels did not correspond to a longer PFS or OS in cancer patients with non-immunotherapy (PFS, HR 1.05, 95% CI 0.63–1.73; OS, HR 1.29, 95% CI 0.48–3.48). Thus, memory CD8(+) T cells might be a promising predictor in cancer patients with immunotherapy.

**Conclusions:**

The host’s overall immune status, and not only the tumor itself, should be considered to predict the efficacy of immunotherapy in cancer patients. This study is the first to show the significant prognostic value of memory CD8(+) T cells in immunotherapy of cancer patients through systematic review and meta-analysis. Thus, the detection of memory CD8(+) T cells has a considerable value in clinical practice in cancer patients with immunotherapy. Memory CD8(+) T cells may be promising immunotherapy targets.

## Introduction

Tumor-infiltrating CD8(+) T cells can predict patient survival and response to immunotherapy in many cancers (1–9). However, it is not clear why some patients with high CD8(+) T cell levels respond significantly to immunotherapy, while others do not. One reasonable explanation is that there are multiple subtypes of tumor-infiltrating CD8(+) T cells with different phenotypes and functions (10–15). Han et al. (14). found three main subtypes of tumor-infiltrating CD8(+) T cells: memory CD8(+) T cells, exhausted CD8(+) T cells, and effector CD8(+) T cells. Exhausted CD8(+) T cells are characterized by low proliferation in response to neoantigen stimulation, progressive loss of effector function, expression of multiple inhibitory receptors such as PD-1, Tim3, and LAG3, and ultimately loss of their tumor-killing effect. Although effector CD8(+) T cells and memory CD8(+) T cells share some similarities at the epigenetic, metabolic, and functional levels, memory CD8(+) T cells can persist for a long time when effector CD8(+) T cells undergo severe contractions. Unlike effector CD8(+) T cells, they are capable of dramatic proliferation after contact with neoantigens and they can maintain the host’s continuous anti-tumor immune state. This suggests that memory CD8(+) T cells play a key role in the sustained tumor killing (16–18). Therefore, it is not comprehensive to predict the efficacy of immunotherapy in patients solely by the number of tumor infiltrating CD8(+) T cells or the level of expression of PD-L1 (19–22). At present, some studies have found that the levels of memory CD8(+) T cells measured by immunohistochemical (IHC) staining can predict the efficacy of immunotherapy in cancer patients, but some studies contradict this conclusion (23). These studies all had insufficient sample size and low universality. Therefore, the objective of the present systematic review and meta-analysis was to determine the prognostic roles of memory CD8(+) T cells in cancer patients who had undergone immunotherapy.

## Methods

The present systematic review and meta-analysis were conducted following the Preferred Reporting Items for Systematic Reviews and Meta Analyses (PRISMA) statement (24).

### Eligibility Criteria

The inclusion criteria were (1) The subject was pathologically diagnosed as a malignant tumor (2), Study subjects must be treated with immune checkpoint inhibitors (3), the relationship between memory CD8(+) T cell levels and PFS or OS was investigated (4), studies containing enough data to estimate the effects HR and 95% CI for PFS or OS. The exclusion criteria included (1): studies without enough data to estimate a HR with a 95%CI (2), studies from review articles, case reports, commentaries and letter.

### Outcome Definitions

Progression-free survival (PFS) is defined as the time from the beginning of immunotherapy to the first disease progression. Overall survival (OS) was defined as the period from the date of tumor diagnosis to the time of death with any cause (25).

### Search Strategy and Study Selection

A systematic literature search was conducted using a combination of key words and Medical Subject Headings (MeSH) terms in the following databases: EMBASE, MEDLINE (PubMed), and Web of Science, to identify eligible articles published before March 2021. The search terms mainly include: stem-like T cell, exhausted T cell, memory T cell, Tumor infiltrating lymphocytes, Cancer and immune checkpoint. Details the retrieval strategy in the Supplementary material.

### Data Extraction

Indeed data were extracted from the included studies using a pilot-tested data extraction form. In addition to the information in **Table 1**, the extracted data included HRs with 95% CIs for PFS or OS.

**Table 1:** Characteristics of included studies.

Study	Country	Treatment regimen	Cancer type	Study type	The phenotype of memory T cells	Selection method of memory T cells	The cutoff between high and low memory T cells level	No. of participants	Outcomes
Tietze JK (2017)	Germany	Ipilimumab/Pembrolizumab	Melanoma	Prospective clinical study	CD45RO(+)	Flow cytometry	Memory T cells ≥ 30% of the total T cells	30	OS
De Coaña YP (2017)	Sweden	Ipilimumab	Melanoma	Prospective clinical study	CD45RA(–) CCR7(–)	Flow cytometry	Divide memory T cells into high and low using Cutoff Finder software.	26	OS
Thommen DS (2018)	Switzerland	Nivolumab	NSCLC	Prospective clinical study	CLCX13(+) PD 1(+)	Flow cytometry	Memory T cells with high expression of PD >1%	21	OS
Wong PF (2019)	USA	Pembrolizumab/Nivolumab/Ipilimumab plus Nivolumab	Melanoma	Prospective clinical study	GZMB(+)	multiplex immunofluorescence	Memory T cells were dichotomized into low and high statuses objectively defined by Joinpoint regression	94	OS
Pender A (2021)_ PFS	Canada	PD-1/PD-LI/PD-1+chemo/PD-L1+chemo	Pan-cancer	Prospective clinical study	CCL5(+)	RNA sequencing	The high and low levels were distinguished by the median of gene expression	31	PFS, OS
IMmotion150	USA	Atezolizumab	Renal clear cell carcinoma	Prospective clinical study	CCL5(+)	RNA sequencing	The high and low levels were distinguished by the median of gene expression	77	PFS
IMmotion150_ Bev	USA	Atezolizumab + Bevacizumab	Renal clear cell carcinoma	Phase II clinical trial	CCL5(+)	RNA sequencing	The high and low levels were distinguished by the median of gene expression	77	PFS
IMvigor210	USA	Atezolizumab	Urothelial carcinoma	Phase II clinical trial	CCL5(+)	RNA sequencing	The high and low levels were distinguished by the median of gene expression	304	PFS
POPLAR	USA	Atezolizumab	NSCLC	Phase II clinical trial	CCL5(+)	RNA sequencing	The high and low levels were distinguished by the median of gene expression	77	PFS

PFS, Progression-free survival; OS, Overall survival.

### Statistical Analysis

We performed meta-analysis to obtain a pooled estimate of the prognostic role of memory CD8(+) T cells using RevMan 5.4. A P-value less than 0.05 was set as indicative of statistical significance. Between-study heterogeneity was measured using the Higgins I^2^ statistic and Cochrane’s Q test (P < 0.10 or I^2^ > 50% was considered indicative of statistically significant heterogeneity) (26). A random effects model was applied if heterogeneity was present. However, the fixed effect model was used in the absence of between-study heterogeneity (P > 0.10 or I^2^ < 50%) (27).

## Results

### Search Results and Study Characteristics

**Figure 1:**
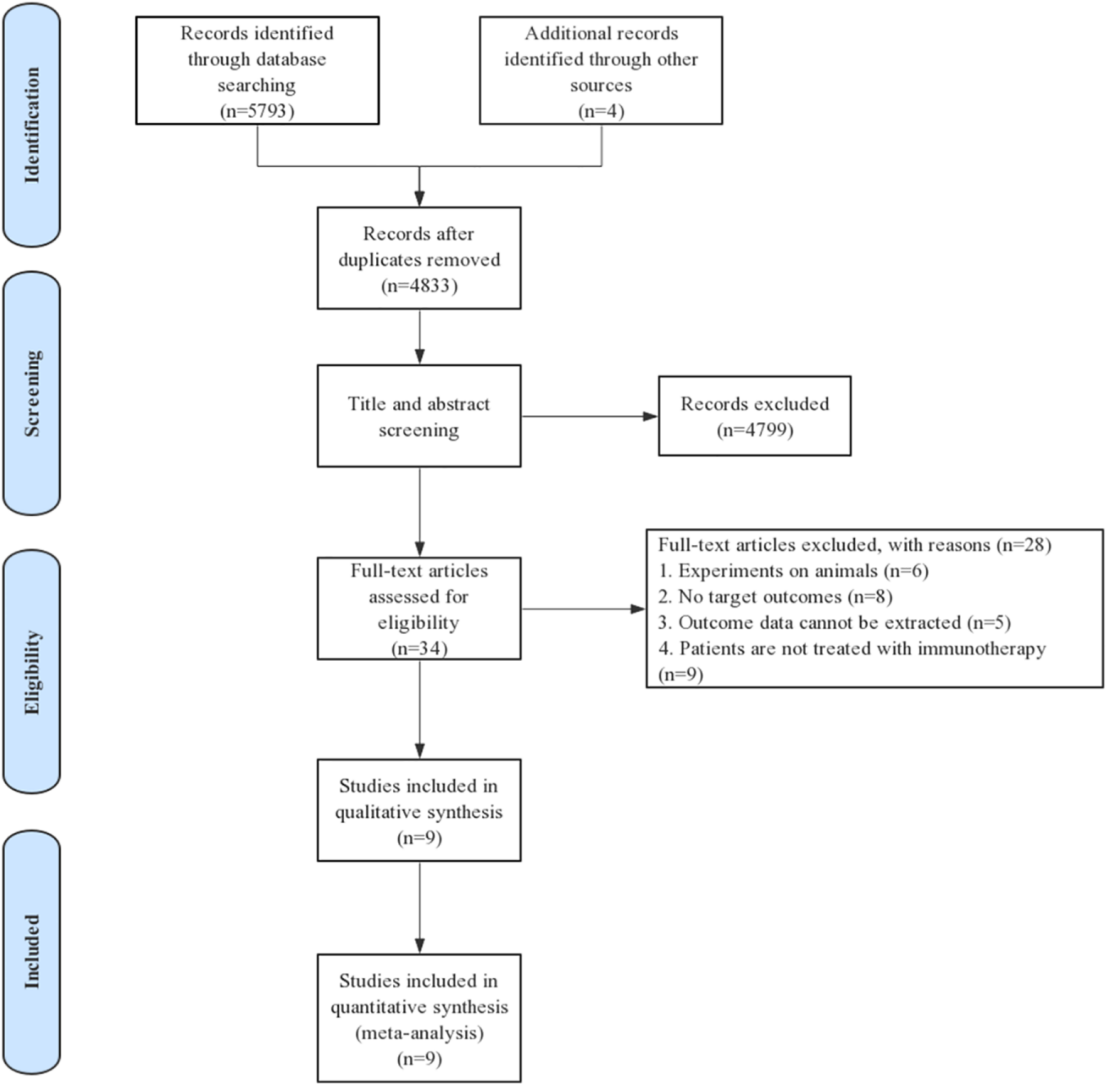
PRISMA flow diagram detailing the search strategy and results.

Eligible studies were identified and selected as shown in **Figure 1**. A total of 5797 articles were for initial evaluation, 4833 studies were eligible after exclusion of duplicates. Abstracts and titles of these studies were reviewed and 4799 studies were excluded. After abstract review, we identified 34 articles for full manuscript review and 28 of these articles were excluded for the reasons delineated in **Figure 1**. Finally, nine studies from six articles (23, 28–32) were included in the final meta-analysis (**Table 1**).

Characteristics for each study are summarized in **Table 1**. All included studies (n = 9) were published from 2017 - 2021. Study sample sizes range from 21 to 304 patients representing an overall total of 743. The studies were conducted in the United States (55.6%, 5/9), Germany (11.1%, 1/9), Sweden (11.1%, 1/9), Switzerland (11.1%, 1/9), and Canada (11.1%, 1/9). The population targeted was cancer patients undergoing immunotherapy.

### Memory CD8(+) T Cells and PFS

**Figure 2:**
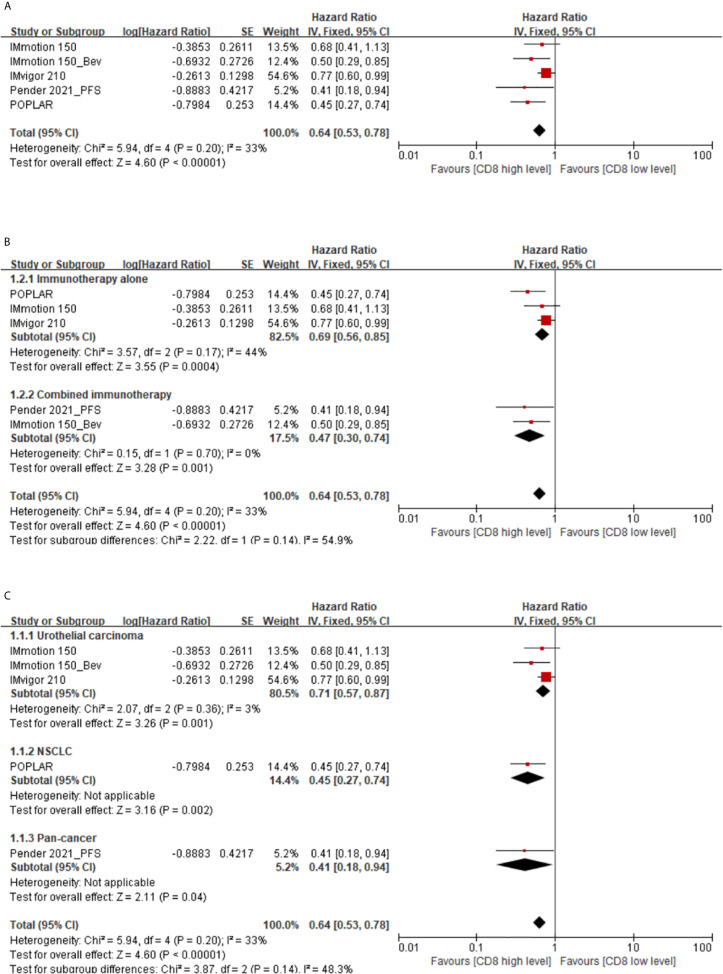
**(A)** Florest plots of the fixed-effects meta-analysis for the effects of memory CD8(+) T cells on PFS. **(B)** Forest plots of the fixed-effects meta-analysis for the effects of the memory CD8(+) T cells on PFS in different treatment regimens. **(C)** Forest plots of the fixed-effects meta-analysis for the effects of the memory CD8(+) T cells on PFS in different types of cancer.

A total of 5 studies supported the prognostic value of memory CD8(+) T cells for PFS in cancer patients treated with immunotherapy. The results showed up high level of memory CD8(+) T cells predicted a better PFS. The pooled HR at the levels of memory CD8(+) T cells was 0.64 (95% CI 0.53–0.78) (high *vs*. low) (**Figure 2A**).

Subgroup analysis showed that high memory CD8(+) T cells levels were indicative of a longer PFS in cancer patients treated with immunotherapy alone (HR 0.69, 95%CI 0.56-0.85) or combined immunotherapy (HR 0.47, 95%CI 0.30–0.74) (**Figure 2B**). Additionally, there also be a prognosis value of memory CD8(+) T cells in urothelial carcinoma (HR 0.71, 95% CI 0.57–0.87), non-small cell lung cancer (NSCLC) (HR 0.45, 95%CI 0.27–0.74), or pan-cancer (HR 0.41, 95% CI 0.18–0.94) patients (**Figure 2C**).

### Memory CD8(+) T Cells and OS

**Figure 3:**
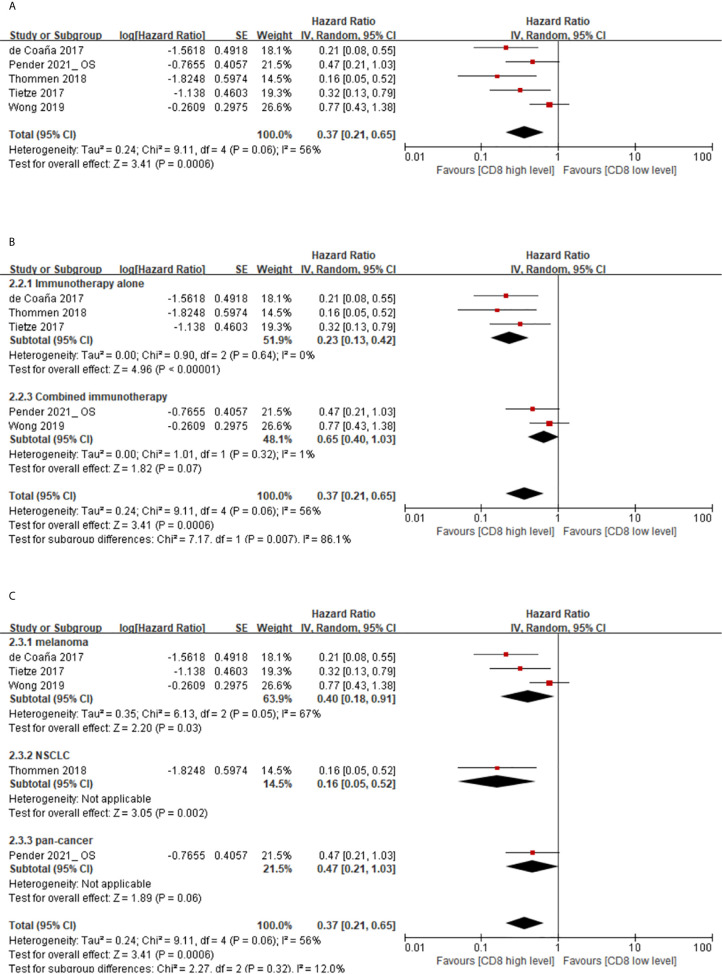
**(A)** Florest plots of the random-effects meta-analysis for the effects of memory CD8(+) T cells on OS. **(B)** Florest plots of the random-effects meta-analysis for the effects of memory CD8(+) T cells on OS in different treatment regimens. **(C)** Florest plots of the random-effects meta-analysis for the effects of memory CD8(+) T cells on OS in different types of cancer.

The association between the levels of memory CD8(+) T cells and OS of cancer patients with immunotherapy was extracted from five studies. The results showed that higher levels of memory CD8(+) T cells correspond to better OS, with pooled HR of 0.37 (95% CI 0.21–0.65) for memory CD8(+) T cell level (high *vs*. low) (**Figure 3A**).

From subgroup analysis according to treatment regimen (high *vs*. low), the HRs were 0.23 (95% CI 0.13-0.42) and 0.65 (95% CI 0.40–1.03) for immunotherapy alone and combined immunotherapy, respectively (**Figure 3B**). Analysis of the subgroup of cancer types showed that the HRs were 0.40 (95% CI 0.18–0.91), 0.16 (95% CI 0.05–0.52) and 0.47 (95% CI 0.21–1.03) for melanoma, NSCLC, and pan-cancer, respectively (**Figure 3C**).

However, there was relatively significant heterogeneity between the main group and the subgroup. Through sensitivity and subgroup analysis, it was found that the heterogeneity was mainly derived from study Wong 2019, and the heterogeneity was significantly decreased in the main group (I^2^ = 0%) and subgroup (I^2^ = 0%) after its elimination. This may be due to the T cells phenotype is GZMB in Wong 2019, which differed significantly from the definition of the T cells phenotype in other studies. In addition, the final conclusions remain the same in the main group (HR 0.30, 95% CI 0.19–0.47) and in the subgroup (HR 0.26, 95% CI 0.14–0.51) after exclusion of Wong 2019. Thus, this analysis confirmed the stability of our results.

### Memory CD8(+) T Cells in the Control Arms

**Figure 4:**
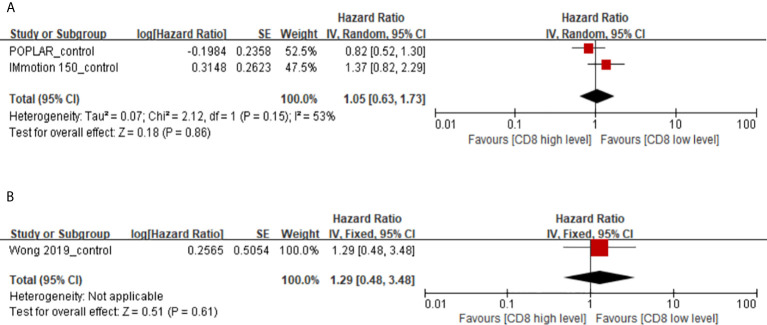
**(A)** Florest plots of the random-effects meta-analysis for the effects of memory CD8(+) T cells on PFS in cancer patients with non-immunotherapy. **(B)** Florest plots of the fixed-effects meta-analysis for the effects of memory CD8(+) T cells on OS in cancer patients with non-immunotherapy.

There are a total of 3 studies with immunotherapy as the experimental group and non-immunotherapy as the control group focused on the predictive value of memory CD8(+) T cells. Our study found that high levels of memory CD8(+) T cells have no effect on PFS and OS in cancer patients with non-immunothrapy (PFS, HR 1.05, 95% CI 0.63–1.73; OS, HR 1.29, 95% CI 0.48–3.48) (**Figures 4A, B**). However, there is significant heterogeneity between the IMmotion 150_control and POPLAR_control groups, which may be due to the difference in baseline treatment regimens between the two groups (Docetaxel in IMmotion 150_control, Sunitinib in POPLAR_control). This may indicate that the poor prognostic value of memory CD8(+) T cell in cancer patients with chemotherapy and targeted therapy.

### Risk of Bias in Included Studies

**Figure 5:**
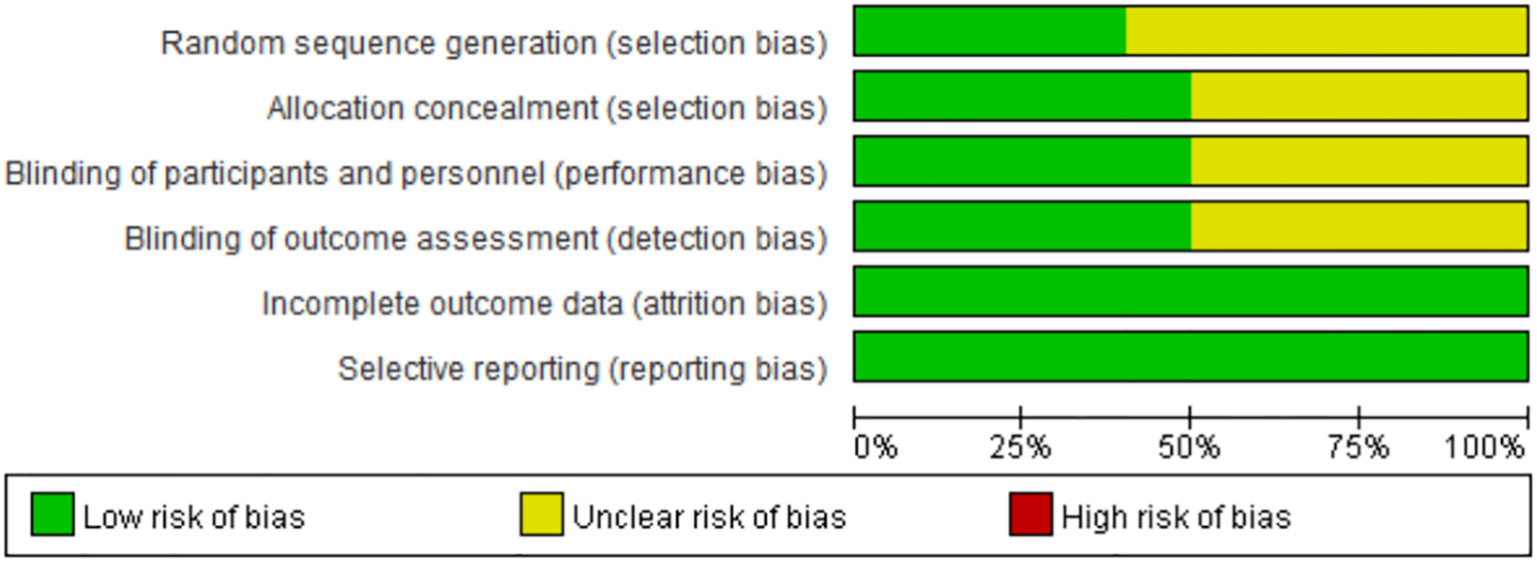
Risk of bias graph: each risk of bias item presented as percentages across all included full reported studies.

Risk of bias were assessed for all included studies (n=9) as shown in **Figure 5**. Since the articles are all prospective clinical trials, the overall risk of bias is relatively low.

### Publication Bias

**Figure 6:**
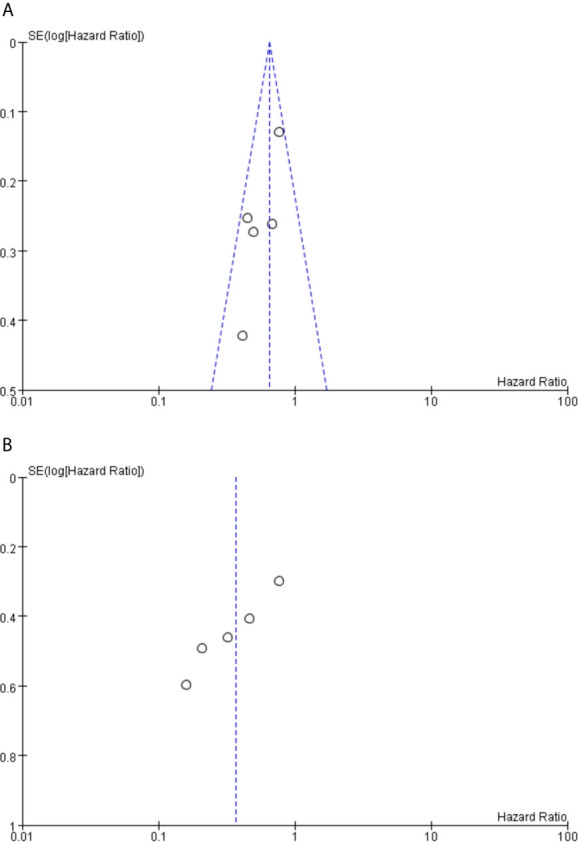
**(A)** Funnel plot analysis of potential publication bias for PFS. **(B)** Funnel plot analysis of potential publication bias for OS.

Funnel plot analysis did not indicate apparent publication bias affecting the HRs for PFS and OS (**Figure 6**).

## Discussion

In the past, tumor immunotherapy focused on T cell immunity, which enabled 20% of patients to enjoy greater clinical benefits (33, 34). In order to further improve the efficacy of immunotherapy and predict the target population, it is necessary to study the roles of T cells of different phenotypes and functions in anti-tumor immunity in depth. In chronic infections, memory CD8(+) T cells have been shown to be a key factor in persistent immune activity. However, its role in anti-tumor immunity is still unclear (35–38). This is the first systematic review and meta-analysis to synthesize nine studies to evaluate the association between memory CD8(+) T cell levels and prognosis of immunotherapy in cancer patients. Our results indicate that high levels of memory CD8(+) T cells in cancer patients with immunotherapy may significantly prolong PFS and OS.

In our study, the indicators of outcome prognosis were PFS and OS. Previous studies reported that higher memory CD8(+) T cell levels predict a worse response to immunotherapy in cancer patients (23). According to our pooled results, cancer patients with high memory CD8 T cell levels showed better PFS (HR 0.64, 95% CI 0.53–0.78) and OS (HR 0.38, 95% CI 0.19–0.73) than those with low memory CD8(+) T cell levels. Furthermore, our study also found a significant predictive effect of memory CD8(+) T cell levels in cancer patients with immunotherapy alone after controlling for interference factors such as chemotherapy, radiation and targeted therapy (PFS; 0.65, 95%CI 0.48–0.89, OS; OS; 0.23, 95% CI 0.13–0.42). One potential explanation for these findings is the influence of memory CD8(+) T cells levels on tumor immunorecognition and tumor immunosuppression (39–41). However, high memory CD8(+) T cells levels did not correspond to a longer PFS or OS in cancer patients with non-immunotherapy (PFS, HR 1.05, 95% CI 0.63–1.73; OS, HR 1.29, 95% CI 0.48–3.48). Thus, memory CD8(+) T cells might be a promising predictor in cancer patients with immunotherapy.

Tumors are a chronic disease, and patients who develop curative immunity to tumor cells are thought to need long-lived T cell immunity, which is mediated by memory CD8(+) T cells (10, 36). A correlation between the numbers of memory CD8(+) T cells and the level of tumor immunity has been firmly established, functional ability of memory CD8(+) T cells also determines the degree of memory CD8(+) T cell-mediated tumor immunity (10, 42, 43). Unlike effector T cells and exhausted T cells, memory CD8 T cells can persist and function throughout host and tumor tissues, making them a potential target for anti-tumor immunity (12, 44). Therefore, patients with high memory CD8(+) T cell levels tend to have better tumor immunosuppressive effects.

Our study also has some specific limitations. First, the number of included studies was limited and the sample size of the included study was insufficient. Second, most of the subjects were still treated with combined immunotherapy, which may cause interference from other factors, such as chemotherapy, radiotherapy, and targeted therapy. In addition, there are also differences in immune checkpoint inhibitors among different studies, and there may be bias errors. Next, there were subtle differences in the definition of memory CD8(+) T cells in different studies, which may be the source of the heterogeneity of the paper. This will also be the direction of further attention and research. Furthermore, our results support prognostic value of memory CD8(+) T cells, however the number of studies in the control arms was relatively limited. Thus, there need more research data to further support the predictive value of memory CD8(+) T cells. Finally. the prognostic value of memory CD8(+) T cells has only been confirmed in lung cancer and urothelial carcinoma, and its value in other types of cancer merits further exploration.

## Conclusion

In recent years, immunotherapy has brought considerable survival benefits for cancer patients, but it has also encountered therapeutic bottlenecks. Currently, biomarkers of the efficacy of immunotherapy, such as TMB, PDL-1 and MSI-H, are all based on tumor cells themselves (19, 45, 46). However, comprehensive prognosis assessment should pay attention to both tumor and host immune status. Therefore, it is urgent to study the immune microenvironment in depth in order to further improve the efficacy of immunotherapy (47).

Based on the above analysis, we can definitively conclude that a higher level of memory CD8(+) T cells corresponds to a better prognosis for immunotherapy in cancer patients, and significant differences were also observed in patients given immunotherapy alone after exclusion of other interfering factors such as chemotherapy, radiotherapy and targeted therapy.

This study is the first to demonstrate that memory CD8(+) T cells play a key role in tumor immunotherapy. More noteworthy is that memory CD8(+) T cells can not only predict the efficacy of patients before immunotherapy, but also may become a promising target for anti-tumor therapy, which may enhance the efficacy of immunotherapy by increasing the content of memory CD8(+) T cells in the immune microenvironment.

## Data Availability Statement

The original contributions presented in the study are included in the article/**Supplementary Material**. Further inquiries can be directed to the corresponding authors.

## Author Contributions

YJ, AT, MP and YW were involved in the conception and design of the study. PW retrieves the database, and ZX extracts the data. YJ and AT were used for statistical analysis. YJ and AT wrote the first draft of the manuscript. JP, PR, RL, and MP wrote parts of the manuscript. All authors contributed to the article and approved the submitted version.

## Funding

National Science Foundation of China (NO. 81770169).

## Conflict of Interest

The authors declare that the research was conducted in the absence of any commercial or financial relationships that could be construed as a potential conflict of interest.
