# Supplementary material for: Prognostic Impact of Memory CD8(+) T Cells on Immunotherapy in Human Cancers: A Systematic Review and Meta-Analysis
Source: Front Oncol. 2021 Jun 25;11:698076. doi: 10.3389/fonc.2021.698076 (PMC8269315; doi:10.3389/fonc.2021.698076)
Supplement: Supplementary file 1 [file DataSheet_1.docx]

**Literature search strategy.**

1. Database: PubMed

Search Strategy:

((((((stem-like T cell) OR (exhausted T cell)) OR (memory T cell)) OR (Tumor infiltrating lymphocytes)) AND ("Neoplasia"[Title/Abstract] OR "Neoplasias"[Title/Abstract] OR "Neoplasm"[Title/Abstract] OR "Tumors"[Title/Abstract] OR "Tumor"[Title/Abstract] OR "Cancer"[Title/Abstract] OR "Cancers"[Title/Abstract] OR "Malignancy"[Title/Abstract] OR "Malignancies"[Title/Abstract] OR "malignant neoplasms"[Title/Abstract] OR "malignant neoplasm"[Title/Abstract] OR "neoplasm malignant"[Title/Abstract] OR "neoplasms malignant"[Title/Abstract] OR "benign neoplasms"[Title/Abstract] OR "neoplasms benign"[Title/Abstract] OR "benign neoplasm"[Title/Abstract] OR "neoplasm benign"[Title/Abstract] OR "Neoplasms"[MeSH Terms])) AND (immune checkpoint)) NOT (review[Publication Type]) (1767).

1. Database: Embase

Search Strategy:

#23 #19 AND #21 AND #22 (1218)

#22 'immune checkpoint':ab,ti NOT 'review':it

#20 'stem-like t cell':ab,ti OR 'exhausted t cell':ab,ti OR 'memory t cell':ab,ti OR 'tumor infiltrating lymphocytes':ab,ti

#19  #1 OR #2 OR #3 OR #4 OR #5 OR #6 OR #7 OR #8 OR #9 OR #10 OR #11 OR #12 OR #13 OR #14 OR #15 OR #16 OR #17 OR #18

#18 'neoplasm, benign':ab,ti

#17 'benign neoplasm':ab,ti

#16 'neoplasms, benign':ab,ti

#15 'benign neoplasms':ab,ti

#14 'neoplasms, malignant':ab,ti

#13 'neoplasm, malignant':ab,ti

#12 'malignant neoplasm':ab,ti

#11  'malignant neoplasms':ab,ti

#10 'malignancies':ab,ti

#9  'malignancy':ab,ti

#8  'cancers':ab,ti

#7  'cancer':ab,ti

#6  'tumor':ab,ti

#5  'tumors':ab,ti

#4  'neoplasm':ab,ti

#3  'neoplasias':ab,ti

#2  'neoplasia':ab,ti

#1  'malignant neoplasm'/exp

1. Database: Web of science

Search Strategy:

# 4 #3 AND #2 AND #1 (2808)

# 3 TS= immune checkpoint

#2 TS= (stem-like T cell OR T cell OR memory T cell OR Tumor infiltrating lymphocytes)

# 1 TS= (Neoplasia OR Neoplasias OR Neoplasm OR Tumors OR Tumor OR Cancer OR Cancers OR Malignancy OR Malignancies OR Malignant Neoplasms OR Malignant Neoplasm OR Neoplasm, Malignant OR Neoplasms, Malignant OR Benign Neoplasms OR Neoplasms, Benign OR Benign Neoplasm OR Neoplasm, Benign)

stem-like T cell OR exhausted T cell OR memory T cell OR
